# Study on Vitamin D Levels in 30 to 40-Year-Old Females With Low Back Pain

**DOI:** 10.7759/cureus.54238

**Published:** 2024-02-15

**Authors:** Nimisha V, Gautham G Nair, Biju C Jose, Angel Abraham

**Affiliations:** 1 Department of Biochemistry, Karnataka Lingayat Education Society (KLE) Academy of Higher Education & Research (KAHER's) Jagadguru Gangadhar Mahaswamigalu Moorsavirmath Medical College and Hospital, Hubballi, IND; 2 Department of Biochemistry, PK Das Institute of Medical Sciences, Ottapalam, IND; 3 Department of Neurological Surgery, PK Das Institute of Medical Sciences, Ottapalam, IND; 4 Department of Biochemistry, Government Medical College Thrissur, Thrissur, IND

**Keywords:** vitamin d levels, 30-40 years females, females, chronic low back pain, vitamin d deficiency

## Abstract

Background: Vitamin D is associated with many skeletal-related processes in the body. A major health problem concerning decreased quality of life is chronic low back pain (LBP). Many studies have proved that chronic pain improves with Vitamin D supplementation. This study aimed to explore the correlation between vitamin D levels and the occurrence of LBP in women aged 30 to 40.

Materials and methods: A case-control study was taken up at PK Das Institute of Medical Sciences wherein 50 cases (women aged 30-34 years who had chronic LBP >3 months) and 50 age-matched controls were included. Frequencies of Vitamin D deficiency, inadequacy, and sufficiency were studied. The t-test for examining statistical significance was employed to compare means. Keeping a 95% confidence interval (p<0.05), the odds ratio was calculated.

Results: Vitamin D deficiency diagnosed when Vitamin D level is <20ng/mL was found in 74% of cases and 48% of controls. Vitamin D levels were not found to be statistically different between cases and controls. The odds ratio was found to be 3.083 (p=0.009), showing that participants with LBP are more expected to be deficient in Vitamin D compared to those without LBP.

Conclusions: Although a higher frequency of Vitamin D deficiency was found in cases compared to controls, the mean value of Vitamin D levels was not found to be statistically different amongst cases and controls. A significant Odds ratio establishes a positive association between LBP and Vitamin D deficiency. The reason could be due to most people being restricted indoors due to COVID-19 restrictions. It is essential to standardize the biochemical analysis of Vitamin D and establish appropriate Vitamin D level ranges specifically tailored for the Indian population.

## Introduction

A vitamin that is known to play a very important part in maintaining good health is Vitamin D which is fat soluble. Various names like sunshine vitamin and antirachitic factor have been given to this vitamin. Vitamin D’s distinctiveness lies in the fact that it is synthesized in our skin when we are exposed to sunlight [1]. Ample sunlight exposure and food items like oily fish (Salmon) are the main sources of vitamin D. Other than problems with the skeletal system, other health issues like increased risk of diabetes mellitus type 2, cancer, and cardiovascular diseases are associated with Vitamin D deficiency [2].

Vitamin D is linked to musculoskeletal function. Low back pain (LBP) is a significant health issue associated with reduced quality of life and elevated expenses for diagnosis and treatment. Disability and absence from work are mainly caused by chronic LBP (pain for more than three months). Three diagnostic categories are used for LBP - radiculopathy, specific LBP, and non-specific LBP. When no specific cause like malignancies, infections, etc., can be pinned to the LBP, it comes under the criteria of non-specific LBP. Non-specific LBP affects over 90% of patients with LBP, and extensive research has indicated a connection between chronic pain and inadequate levels of Vitamin D. Vitamin D plays an indispensable part in controlling inflammatory cytokines, which tend to be elevated in chronic pain conditions [3]. Additionally, some studies have shown that Vitamin D supplementation can improve chronic pain [4]. It has also been claimed that any pain continuing for more than six months also called chronic pain from any site will respond to pain therapy only when there is adequate Vitamin D in the body [5].

Vitamin D helps in maintaining serum calcium levels and in specialized muscle protein production. Serum Calcium which is important for muscle contraction and protein synthesis is increased by Vitamin D. The Vitamin D receptor (VDR) in the nucleus has a regulatory role in several Vitamin D actions. These VDRs are found in all tissues of the body [2,6]. A direct effect of Vitamin D on muscles helps in improving musculoskeletal function. Out of the slow and fast types of muscle fibers, it is the Type II fast twitch muscle fibers that are the first to come into action to avert a fall [7].

The interesting point is that Vitamin D inadequacy has been found to cause type II muscle atrophy. A study done in 1979 clearly states that Vitamin D deficiency causes atrophic changes like increased interfibrillar space, necrosis, etc. [8]. It has been found that as a person grows old the risk for deficiency in Vitamin D levels increases significantly. This is evident from the fact that in the United States, around 20%-100% of older people are suffering from Vitamin D deficiency [9].

As the women between the age group 30 and 40 years are bound to home, they have minimal exposure to sunlight. We know that sunlight is a major factor contributing to the production of vitamin D in our body. Hence, factors like decreased skin synthesis of Vitamin D, more time spent indoors than outdoors, and decreased intake of Vitamin D-rich food contribute to Vitamin D deficiency. The fact worth noting is that tissues like fat and muscle cannot store cholecalciferol till its level in the blood is above 20-50ng/mL. Only when it crosses this level, Vitamin D can work without being affected by amounts coming from diet and exposure to sun [10]. Various inflammatory pathways related to chronic pain are known to be affected by Vitamin D. This could be a reason why Vitamin D supplementation helps in improving muscle strength. The discovery of VDRs all through the human body has brought more importance to the study of health conditions related to Vitamin D. Many health problems like hypertension, diabetes, cancer, etc., are now linked with Vitamin D deficiency [6,11]. In India, an average prevalence of 70%-100% has been found in the general population [12]. In a study, the prevalence of Vitamin D deficiency among rural women was found to be 69.94% [13]. As a result, it is the need of the hour to examine the role of Vitamin D in warding off various health issues as well as to study the part played by Vitamin D supplementation in treating those conditions in women. This study was done to study the prevalence of Vitamin D deficiency among women aged 30-40 years who have chronic LBP compared to those who do not complain of the same. This will help in analyzing the association between Vitamin D and chronic LBP.

## Materials and methods

This was a case-control study done at PK Das Institute of Medical Sciences, Vaniyamkulam, Kerala, India, from September 2021 to October 2021 and was started after getting approval from the Institutional Ethics Committee) (IEC Number: IEC/04/35/21). This study was supported and funded by ICMR. According to the study done by Garg et al., Vitamin D deficiency prevalence among rural women was found to be 69.94% [13]. Our study area is Vaniyamkulam which is a village in Palakkad district in Kerala. Test size was calculated utilizing the technique 4pq/L^2^ (where p refers to the prevalence of Vitamin deficiency, q is 100-p, and L is the total permissible error which was taken as 20%) (95% confidence interval) and a value of 43 was arrived at. To this non-response rate of 10% was added to arrive at 47.3. This was rounded off to 50. Due to the case-control nature of this investigation, a 1:1 ratio was taken and 50 age-matched women who came to the Biochemistry lab for a general body check-up and did not have LBP were taken as controls.

Exclusion criteria

Those who had specific LBP, radiculopathy, heavy occupational physical activity, lumbar surgery, kidney diseases, diseases of the liver, and thyroid, and those taking Vitamin D supplementation were excluded from the study.

Inclusion criteria

Women between the ages of 30 and 40 who had been experiencing chronic LBP for at least three months were included in the analysis. Fifty healthy women of similar age were used as controls. An informed consent for the study was collected from the participants.

Sample collection

The venous blood sample was taken under aseptic precautions. Blood samples of selected participants were collected in the Biochemistry laboratory. The parameter used for this study was the level of 25(OH) Vitamin D and it was estimated by using the Beckman Coulter Access 2 Machine (Beckman Coulter Inc., Brea California). The test method used is Chemiluminescence Immunoassay (CLIA). This assay determines a total of 25 hydroxyvitamin D levels in the sample using paramagnetic particle, chemiluminescent immunoassay, and shows a linearity and measuring range of 7.0ng/mL to 120ng/mL [14].

Internal quality was maintained by running the Internal Quality Assurance Program by RANDOX (Randox Laboratories Ltd., UK) at two levels every 24 hours. The values were checked for being within the 2 Standard deviation range, presence of positive or negative bias, errors according to Westgard rules of quality control, and if there was a need for calibration. External quality control was maintained using the External Quality Assurance Program by BIORAD (Bio-Rad Laboratories, Inc. USA). Calibration for this test was done every 28 days using Access 25(OH) Vitamin D Total Calibrators. The limit of the blank, the limit of detection, and the limit of quantitation were 1.5ng/mL, 2.0ng/mL, and 7.0ng/mL, respectively [15].

Statistical analysis

After entering and cleaning the data, an Excel sheet was created from the results. It was then exported for statistical analysis in SPSS version 21 (IBM Corp., Armonk, NY). Descriptive statistics were used to study the mean and standard deviation. The study examined the prevalence of vitamin D deficiency, inadequacy, and adequacy in both cases and controls. Vitamin D mean concentrations were compared between cases and controls using an independent t-test to identify any notable distinctions. This was assessed with a 95% confidence interval, and the p-value of <0.05 indicated statistical significance. To study the association between Vitamin D levels and chronic LBP, odds ratio was calculated to get the risk estimate with a 95% Confidence Interval, and a p-value of <0.05 was taken as statistically significant.

## Results

This study was done among 50 cases who were females with low back pain within 30-40 years of age and 50 healthy controls who were women without low back pain within 30-40 years of age. The cases had a mean age of 36 years, while the controls had a mean age of 33. In the case group, the mean Vitamin D level was determined to be 19ng/mL, in contrast to the control group where it measured at 21.9ng/mL.

A categorization of 30ng/mL as an acceptable level, 20-29ng/mL as a suboptimal/inadequate level, and below 20ng/mL as a deficient level was employed to assess the prevalence of vitamin D deficiency, insufficiency, and sufficiency. In this study, vitamin D deficiency was observed in 74% of the 50 patients and 48% of the controls. Vitamin D inadequacy was noted in 16% of the cases and 32% of the controls. Adequate vitamin D levels were detected in 10% of the cases and 20% of the controls (Table 1).

**Table 1:** Comparison of cases and controls with regards to frequencies of Vitamin D level categories among controls.

	Cases (%)	Control (%)
Deficiency (<20ng/mL)	74.0	48.0
Inadequate (20-29ng/mL)	16.0	32.0
Sufficient (>30ng/mL)	10.0	20.0

On using an independent t-test to know the statistical significance of the difference between the mean values of Vitamin D levels among cases and controls, it was found that the p-value was 0.132. Hence, Vitamin D levels in cases and controls were not found to be statistically different (Table 2).

**Table 2:** Independent t-test to know the statistical significance of the difference between the mean values of Vitamin D levels among cases and controls. **p-value - Not statistically significant as p > 0.05

Independent sample t-test	Group	N	Mean	Std. deviation	t-value	P-value
Vit D levels	case	50	19.000	8.3005	1.518	0.132**
	control	50	21.920	10.7700		

The odds ratio was calculated to get the risk estimate and it was found to be 3.083 with a 95% confidence interval and p-value of 0.009. Hence, the association between low back pain and Vitamin D Deficiency was found to be statistically significant (Table 3).

**Table 3:** Odds ratio calculation to get the risk estimate. *p value < 0.05, hence statistically significant

Vitamin D levels	Case (with low back pain)	Control (without low back pain)	OR (95% CI)	B	P-value
1 (Vitamin D Deficient)	37	24	3.083 (1.33 – 7.149)	1.126	0.009*
2 (Vitamin D not Deficient)	13	26		2.172	

## Discussion

Vitamin D levels were not observed to be significantly different in the current investigation among cases were women aged 30-40 years who had LBP and controls who were women aged 30-40 years who did not have back pain (p-value 0.132(>0.05), using independent t-test). On the other hand, a noteworthy odds ratio of 3.083 (p-value 0.009, 95% Confidence Interval) was attained, signifying that individuals afflicted by LBP had vitamin D deficiency in comparison to those without LBP. As a result, a positive association between these two conditions is confirmed. Hence Vitamin D supplementation can be considered in women aged 30-40 years who come with complaints of chronic LBP. Also, Vitamin D levels should be measured in patients who come with such complaints, so that follow-up can be done regarding the same. Similarly, an extensive meta-analysis conducted by Zadro et al. concentrating on the relationship between Vitamin D and LBP, likewise uncovered a positive connection between LBP and lack of Vitamin D. The amalgamated results from 19 studies indicated that lack of Vitamin D was more prevalent among people with LBP than in those who did not experience any back pain. The association between LBP and Vitamin D deficiency was found to be statistically significant among women compared to men where it was not found to be statistically significant [16]. In a review observational examination done by Gokcek and Kaydu, it was found that decreased degrees of Vitamin D were related to increased severity of LBP [17]. In this study, there was no notable distinction observed in terms of Vitamin D levels between the case and the control group. This seems to have opened a Pandora’s box in front of us. In the cases, the mean value was found to be 19ng/mL compared to controls where it was found to be 21.9ng/mL, both these values coming under deficient and inadequate levels. In the Indian population, it is noted that the average prevalence of Vitamin D deficiency is 70%-90% [12]. In that case, this study could not mark a difference between cases and controls when the prevalence is so high. Added to this is that this study was done from September 2021 to October 2021, when the country was still limping back to normalcy from COVID-19. For almost two years people have been indoors which might have affected the outcome of this study. Without sufficient sunlight exposure in these years, it will not be a surprise that Vitamin D deficiency might have established itself as a pandemic, the silent presence of which will be evident in the years to come due to the long-term effects that Vitamin D Deficiency has on the human body. Large-scale population studies that analyze the levels of Vitamin D in different population subsets based on age and gender are required to bring the black cat out of the darkroom so that actions can be taken at the state and national levels to address the issue of Vitamin D deficiency.

Some questions that arose when studying the consequences of this study are: 1. Is the scope of Vitamin D levels appropriate for the Indian population as a major chunk of the population was found to be deficient in Vitamin D levels? 2. Is the assay technique accurate and precise? In the review article by Selvarajan et al., 2,998 articles were reviewed, and it was found that the Lack of vitamin D is far-reaching among the sound Indian populace regardless of how much time was spent outside in daylight. The typical Vitamin D level among the Indian population is found to be below 20ng/mL. Although the classification given by endocrine society as sufficient, inadequate, and deficient is being widely accepted, as most of the world population is found to be in the deficiency range the basis of this classification is being scrutinized. It is suggested in their article that studies need to be done to establish a classification of Vitamin D levels for the Indian population [12].

A study done to gauge the link between bone mineral density, parathyroid hormone, and Vitamin D, found that bone health is dependent on PTH levels and not on Vitamin D levels alone [18]. Even in our study, including PTH levels might have given a better picture regarding LBP among cases. The classification of Vitamin D status as deficient, insufficient, and adequate is based on skeletal effects with the consistent increase in PTH and decrease in calcium absorption from the intestine. It is found that when serum levels reach 30ng/mL, intestinal calcium absorption is at its highest, and PTH levels start decreasing. A safe level of Vitamin D is considered 30-100ng/mL [19].

It is known that Vitamin D undergoes two hydroxylation processes in the body, first to form 5 hydroxyvitamin D in the liver and second to frame 1,25 dihydroxy Vitamin D in the kidney. Of these two metabolites, 25(OH)D is the principal metabolite to know Vitamin D status as 1,25 (OH)2D is viewed as either in the ordinary range or increased due to a secondary increase in serum PTH due to Vitamin D deficiency. Also, the half-life of 25(OH)D is two to three weeks compared to 1,25 dihydroxy vitamin D which is only two to three hours [20]. A very important point by Spedding et al. is that the optimal cut-off for Vitamin D might be dependent on the disease in question [21]. There are various methods routinely used for Vitamin D quantification like liquid chromatography-mass spectrometry (LC-MS) or tandem mass spectrometry, chemiluminescence immunoassays (CLIA) radioimmunoassays (RIA), and (LC-MS/MS), LC with UV detection, most widely being utilized are RIA, and CLIA. The gold standard method for the estimation of Vitamin D is LC-MS/MS. The review article by Stokes on Analytical Methods for Quantification of Vitamin D discusses various factors responsible for discrepancies noted in Vitamin D measurement. It is stated that there are many sources of variation in Vitamin D analysis like tight binding of Vitamin D binding protein, matrix effects, and co-elution of interfering substances. It has been stated that the chance of an assay giving a lower than actual reading is much more than that assay giving a higher than actual reading. This was found because most of the commercial assays gave results lower compared to the gold standard test that is LC-MS. This has resulted in overtreatment with Vitamin D supplements in the world [22].

An upward trend is being noted amongst increasing awareness regarding Vitamin D deficiency in India. This goes without saying that, the 25(OH)D test is still unaffordable for most Indians. Here many questions arise like if it is necessary to get everybody’s Vitamin D status checked. Is it necessary to treat subclinical cases? These do not seem to be economical. As suggested by the authors of the review article, food fortification seems to be one of the solutions for this. A minimum of 45 minutes of direct sun exposure to the bare face, legs, and arms is suggested for adequate vitamin D. The problem with this statement lies in the fact that those who must work outdoors seem to be the only people who can get this type of exposure. The rest of us must hide behind the veil of social and religious norms which makes most of us dress from neck to toe [19]. Added to this was the long indoor stay during the COVID-19 pandemic. Low levels of Vitamin D lead to an increase in serum PTH which in turn leads to bone resorption. Hence for good skeletal health, we need to keep our Vitamin D at optimum levels.

In our research, we identified a favorable connection between vitamin D and lower back pain. Nevertheless, the absence of a noteworthy distinction in vitamin D levels between the case and control groups implies that this merely scratches the surface regarding the widespread occurrence of vitamin D insufficiency. A bigger issue lies behind this veil of the term “Vitamin D deficiency,” which includes not only the superficial causes of inadequate sun exposure and diet but also deeper issues like non-standardized lab techniques, complicated assays, and inappropriate population ranges. We have a long way to go before we say that the Vitamin D level and range are accurate and precise for a given individual in the given population.

The limitation of this study was that this was a cross-sectional study with a limited sample size and population. In our study a population subset of women aged 30-40 years was taken, hence findings of the same cannot be projected to the whole population. This study adds to the evidence that Vitamin D deficiency is associated with chronic LBP and hence provides more insight into its causation. A longitudinal study where, in different population subsets, cases are studied after giving Vitamin D supplementation would have converted the limitation of this study to the strength of this study. As was mentioned before serum PTH has a direct relationship to bone density, in our study too including PTH levels might have done justice in understanding the occurrence of LBP among Vitamin D deficient cases. To bring the Vitamin D deficiency pandemic out in the public eye, large population studies must be done across states and countries to know the levels of Vitamin D using standardized methods in the respective populations. These Vitamin D levels need to be categorized based on different population subsets. Doing so will give an exact picture of Vitamin D deficiency in a particular population subset.

## Conclusions

This study was done to study the association between LBP and Vitamin D levels among cases and controls who were 30 to 40-year-old females with and without LBP, respectively. Among 50 cases, Vitamin D deficiency was found in 74% of cases compared to 48% of controls. No statistically significant difference was found in levels of Vitamin D among cases and controls. A statistically noteworthy positive association (OR-3.083, 95% confidence Interval, p=0.009) was established between Vitamin D deficiency and LBP. Hence Vitamin D levels can be estimated in women aged 30 to 40 years who come with complaints of non-specific chronic LBP. Also, Vitamin D supplementation might help in treating cases of non-specific LBP. Adding serum PTH levels and newer follow-up studies that study cases before and after Vitamin D supplementation are required to give a better answer. This study's findings might have been influenced by the fact that most participants spent the previous two years inside because of COVID-19 regulations. We also need to work on a new normal range for the Indian population as most of the population is coming under the category of Vitamin D deficiency. Analytical standardization is also required to reduce variability and reporting of lower than true values. The results of the study seem to have uncovered a bigger problem which might be staring at the face of humanity, that is explosive number of Vitamin D deficient people due to COVID-19 restrictions. Standardizing both the biochemical assessment of Vitamin D and the suggested Vitamin D level ranges is of utmost importance.
